# A Resource for Discovering Specific and Universal Biomarkers for Distributed Stem Cells

**DOI:** 10.1371/journal.pone.0022077

**Published:** 2011-07-19

**Authors:** Minsoo Noh, Janet L. Smith, Yang Hoon Huh, James L. Sherley

**Affiliations:** 1 School of Pharmacy, Ajou University, Suwon, South Korea; 2 Programs in Regenerative Biology and Cancer Biology, Adult Stem Cell Technology Center, Boston Biomedical Research Institute, Watertown, Massachusetts, United States of America; City of Hope National Medical Center and Beckman Research Institute, United States of America

## Abstract

Specific and universal biomarkers for distributed stem cells (**DSCs**) have been elusive. A major barrier to discovery of such ideal DSC biomarkers is difficulty in obtaining DSCs in sufficient quantity and purity. To solve this problem, we used cell lines genetically engineered for conditional asymmetric self-renewal, the defining DSC property. In gene microarray analyses, we identified 85 genes whose expression is tightly asymmetric self-renewal associated (**ASRA**). The ASRA gene signature prescribed DSCs to undergo asymmetric self-renewal to a greater extent than committed progenitor cells, embryonic stem cells, or induced pluripotent stem cells. This delineation has several significant implications. These include: 1) providing experimental evidence that DSCs *in vivo* undergo asymmetric self-renewal as individual cells; 2) providing an explanation why earlier attempts to define a common gene expression signature for DSCs were unsuccessful; and 3) predicting that some ASRA proteins may be ideal biomarkers for DSCs. Indeed, two ASRA proteins, CXCR6 and BTG2, and two other related self-renewal pattern associated (**SRPA**) proteins identified in this gene resource, LGR5 and H2A.Z, display unique asymmetric patterns of expression that have a high potential for universal and specific DSC identification.

## Introduction

A long-standing challenge in mammalian stem cell biology is discovery of specific biomarkers for non-embryonic stem cells [1]–[3]. Non-embryonic stem cells include the diverse stem cells that emerge in fetal or post-natal development to sustain both somatic and germinal tissues. Of the many names used for non-embryonic stem cells, including, but not limited to, adult stem cells, tissue-specific stem cells, germline stem cells, and somatic stem cells, none give a comprehensive exact description. We recently introduced the name “distributed stem cells (**DSCs**)” for this purpose [4]. “Distributed” embodies the common aspect of all non-embryonic stem cell types to retain a limited, specific distribution of the pluripotent developmental potential of embryonic epiblasts.

The ability to identify and quantify DSCs directly would revolutionize tissue cell research and cell-based medicine. This achievement requires DSC biomarkers of superior specificity. Until recently, only a handful of proteins had been described that were even preferentially expressed in DSCs. However, most of these “stem cell markers” are low-specificity DSC biomarkers, because they are also significantly expressed by more abundant lineage-committed progenitor cells produced by DSCs [1], [2], [5]. This generally poor success in discovering biomarkers that are expressed only in DSCs led to the approach of DSC identification by the *absence* of lineage-specific proteins expressed by cells committed to differentiation [1]. However, because of its technically challenging serial cell sort analyses and inherently poor specificity, this lineage-negative biomarker approach does not provide an effective means for DSC quantification.

The first example of a potentially exclusive DSC biomarker is the product of the *Lgr5 gene*, a member of the leucine-rich-repeat containing G-protein-coupled receptor gene family. Fluorescent reporter protein genes knocked-in to the mouse *Lgr5* locus identified cells with stem cell character in intestinal crypts, colonic pits, and hair follicles [6]–[9]. Antibodies against the human Lgr5 protein also identify a rare population of cells in the stem cell niche regions of human intestinal crypts and colonic pits [10]. A related gene family member, *Lgr6*, has a similar phenotype in mouse knock-in studies [11]. Whether Lgr5 and Lgr6 will prove to be universal biomarkers for DSC depends on their evaluations in DSCs in other tissues [12]. Recent reports also highlight uncertainties regarding their identification of DSCs *versus* more numerous committed progenitor cells in intestinal crypts [13].

Several properties of DSCs contribute to their past poor history of exclusive biomarker discovery. Foremost, they constitute a rare fraction of tissue cells. In many tissues, their fraction is estimated to be less than 1 in several thousand and in some as low as 1 in 100,000 [14]. Their defining characteristic, asymmetric self-renewal, is a major barrier to their detection, isolation, and expansion [3], [15]. DSCs simultaneously produce non-stem differentiating progeny cells while self-renewing without loss of stem cell capacity. Because of asymmetric self-renewal, in culture, cell populations initially enriched for DSCs rapidly decline in DSC fraction because of the cells' own asymmetric production of differentiating cells [15]–[18]. Thus, DSCs are highly refractory to production in the quantity and purity needed for effective biomarker discovery.

Herein, we report a new general approach for discovery of specific and universal DSC biomarkers. The approach is grounded in the precept that the defining function of DSCs, asymmetric self-renewal, is a high-fidelity DSC-specific property [3], [19]. It follows that some biomarkers specific to asymmetric self-renewal will also be specific to DSCs *per se*. This approach is feasible because of the availability of genetically engineered cell lines that recapitulate asymmetric self-renewal division under experimental control [17], [20], [21]. We report the use of these model cell lines to identify genes whose mRNA expression level differs significantly between symmetrically self-renewing cells and asymmetrically self-renewing cells. Whether up-regulated or down-regulated during asymmetric self-renewal with respect to symmetric self-renewal, the 85 genes so identified are called “asymmetric self-renewal associated” (**ASRA**) genes. Our findings show that ASRA genes are a unique set of genes for detection of DSC asymmetric self-renewal and a new resource for discovery of specific and universal DSC biomarkers.

## Results

### Discovery of an ASRA gene subset

Previously, we established the use of genetically engineered cell lines to define molecular pathways responsible for asymmetric self-renewal [17], [20], [21], [22]–[26]. The self-renewal pattern of the cell lines (asymmetric *versus* symmetric) is under investigator control. Both temperature-controlled [22]–[24] and Zn-controlled models [17], [20], [21], [25], [26] have been described with similar conditional asymmetric self-renewal properties.

**Figure 1:**
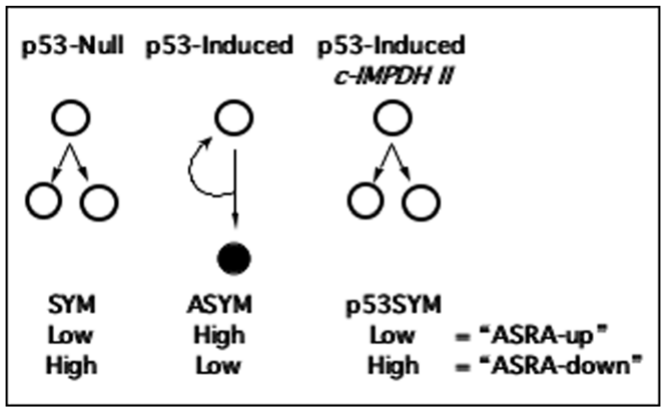
Genetically engineered p53-inducible cell line strategy for discovery of asymmetric self-renewal associated (ASRA) genes. All cell lines are maintained in ZnCl_2_-supplemented medium. Vector control, **p53-Null** cells exhibit symmetric self-renewal (**SYM**) under this condition. The SYM state is characterized by continuous cell divisions that produce two equivalent cycling cells (**open circles**). P53-inducible cells exhibit asymmetric self-renewal (**ASYM**) in response to **p53** expression **induced** by ZnCl_2_-supplementation. The ASYM state is characterized by continuous cell divisions that produce one cycling cell and one G1/S-arrested cell (**closed circle**; **curved arrow** indicates continuous asymmetric divisions by cycling stem-like cells). **c-IMPDH II**-expressing cells induce p53 as well, but maintain symmetric self-renewal (**p53SYM**). **“ASRA-up”** genes are defined as those that were undetectable or produced at **low** levels in both SYM and p53SYM states, but showed **high** levels of expression in the ASYM state. Genes with the reciprocal expression pattern are called **“ASRA-down”** genes.

The Zn-controlled engineered cells were used for these studies [26]. These lines are p53-null murine fetal fibroblasts engineered to conditionally express normal levels of the wild-type murine p53 protein from a p53 cDNA controlled with a modified human metallothionein promoter [17], [25], [26]. In maintenance culture medium (*i.e.*, Zn-free), the conditional p53 minigene is off, and the cells undergo symmetric self-renewal. In ZnCl_2_-supplemented medium, normal levels of wild-type p53 protein are produced, and the cells switch to asymmetric self-renewal (Fig. 1, ASYM). During asymmetric self-renewal, a subpopulation of cells divides continuously (*i.e.*, DSC-like cells), but produces a G1/S-arrested daughter cell every ∼24-hour division cycle (Fig. 1, closed circle). Control p53-null cell lines, co-derived by stable transfection of the human metallothionein promoter vector without a p53 cDNA insert, retain symmetric self-renewal in ZnCl_2_-supplemented medium (Fig. 1, SYM) [17], [21], [25], [26].

Alone, the p53-inducible cell lines were not ideal for discovery of genes whose expression varied specifically with changes in self-renewal pattern, because p53 is also an activator of many cellular systems in addition to those associated with asymmetric self-renewal. We devised a filter to discriminate ASRA genes based on our earlier discovery of a p53-dependent pathway that regulates self-renewal pattern in the engineered cell lines. The p53-dependent down-regulation of the type II inosine-5′-monophosphate dehydrogenase (IMPDH II; EC 1.2.1.14) is required for asymmetric self-renewal by the engineered cell lines [21]–[23], [25] and DSCs [15], [18], [27]–[29]. IMPDH II is the rate-limiting enzyme for cellular guanine ribonucleotide biosynthesis. Previously, we established congenic derivatives of the Zn-controlled p53-inducible cell lines that constitutively expressed an IMPDH II transgenic cDNA (“**c-IMPDH II**”) [25]. The c-IMPDH II lines retain Zn-inducible p53 expression, but they no longer shift from symmetric self-renewal to asymmetric self-renewal (Fig. 1, **p53SYM**) [21]. P53-responsive genes like *p21waf1*, *bax*, and *mdm2* still show induced expression in the c-IMPDH II lines, but the cells maintain symmetric self-renewal [21], [25]. Control cells co-derived with the expression vector without the IMPDH II cDNA insert retain the Zn-inducible shift from symmetric self-renewal to asymmetric self-renewal [25].

Fig. 1 illustrates the complete experimental system employed for ASRA gene discovery based on congenic p53-null (SYM), p53-inducible (ASYM), and c-IMPDH II (p53SYM) cell lines all grown in Zn-supplemented medium for 48 hours (*i.e.*, 2 cell generations; see *Materials and Methods*). Based on this design, ASRA genes were defined as those whose expression was up-regulated or down-regulated in the ASYM state with respect to the SYM and p53SYM states, which were required to have similar expression levels. When a gene's expression is different in the ASYM state compared to the SYM, the change could be due to induced p53 expression *per se*, the acquisition of the ASYM state, down-regulation of IMPDH II, or any combination of these three known effects. A p53-responsive gene that does not require asymmetric self-renewal for its change will show a similar expression change in the p53SYM state. The same is true for IMPDH II-responsive genes that do not require the asymmetric self-renewal state. This is because IMPDH II expression in the p53SYM state is also elevated with respect to the SYM state [25]. In contrast, ASRA genes show lesser or no change in the p53SYM state (*i.e.*, their expression level will be similar in SYM and p53SYM states), because, in principle, their change also requires acquisition of the state of asymmetric self-renewal.

**Figure 2:**
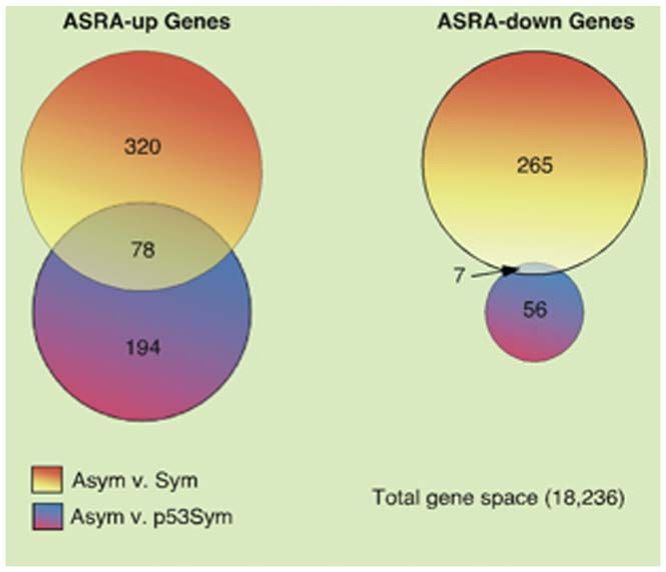
Bioinformatics strategy for discovery of the 85 gene ASRA signature. ASRA genes (**“ASRA-up”** and **“ASRA-down”**) are the respective unions of two intersection sets. The ASRA-up intersection was defined as genes that were up-regulated in the ASYM state with respect to the SYM state *AND* up-regulated in the ASYM state with respect to the p53SYM state. The ASRA-down intersection was defined as genes that were down-regulated in the ASYM state with respect to the SYM state *AND* down-regulated in the ASYM state with respect to the p53SYM state (See also self-renewal pattern definitions in Fig. 1).

Operationally, first we identified genes whose expression was either significantly up-regulated or significantly down-regulated in the ASYM state with respect to either the SYM state (A comparison) or the p53SYM state (B comparison) (Fig. 2; see *Materials and Methods*). The intersection set, ASYM *v.* SYM∩ASYM *v.* p53SYM, for respective up-regulated and down-regulated genes yielded 85 genes that met the ASRA criteria (Fig. 2; Table S1).

**Figure 3:**
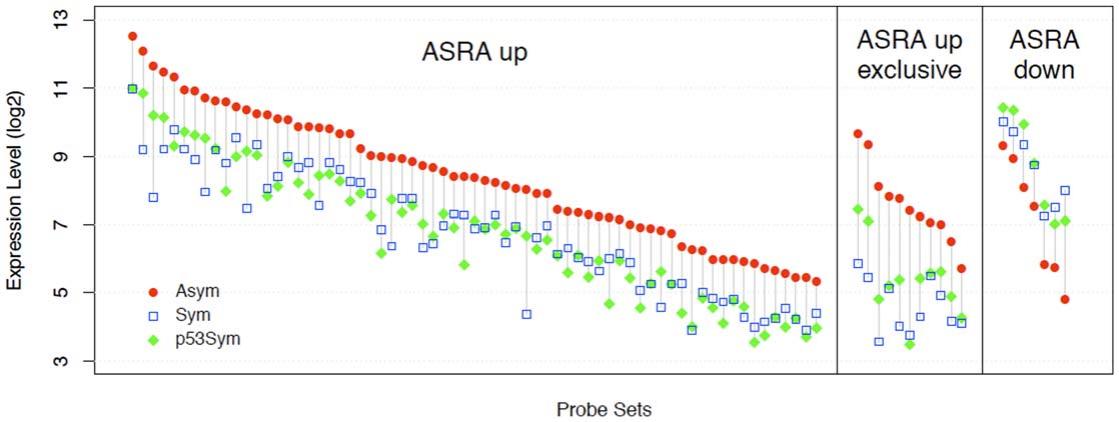
Quantitative assessment of the ASRA gene signature. The log2 expression levels of the microarray probe sets defining the three main quantitative categories of the ASRA gene signature (**ASRA up**, **ASRA up exclusive**, **ASRA down**) are graphed for each of the three compared self-renewal states. Data for each gene category (**x-axis**, **Probe Set**) are ranked-ordered based on the ASYM expression level (**y-axis**). A vertical line connects the ASYM and SYM expression levels.

Fig. 3 provides a quantitative comparison of up-regulated and down-regulated ASRA genes ranked by their average microarray signal intensity for the ASYM state. Only 7 ASRA genes were down-regulated (Table S1, “ASRA down” genes) in asymmetrically self-renewing cells. Among the up-regulated ASRA genes (Table S1, “ASRA up” genes) showing the highest degree of induction (4-fold to 23-fold) are previously described p53-responsive genes (*e.g.*, *Trp53inp1*, *Cdkn1a*, *Ccng1*, *Mdm2*). Twenty up-regulated ASRA genes were scored as “absent” in at least one of the two SYM states (see Table S1). The eleven that scored as “absent” for expression in the SYM state (*4833427G06Rik*, *Sulf2*, *Khdrbs3*, *Cdkn1a*, *Eda2r*, *Rftn1*, *2410016O06Rik*, *3110039M20Rik*, *F11r*, *Myo1b, and Foxs1*), were named “exclusive” ASRA genes. Exclusive ASRA genes were also in the top half for degree of induction in the ASYM state (compared to their SYM background intensity). Nineteen ASRA genes are predicted to encode membrane proteins, including 2 exclusive ASRA genes (*Eda2r* and *F11r*; see Table S1).

Although ASRA genes were selected as the ideal candidates for specific and universal DSC biomarkers, other genes whose expression changes between the three states of self-renewal are also of interest. These members of the larger group of self-renewal pattern associated (**SRPA**) genes, which number 845, are also listed in Table S1. They are defined as A_up: up-regulated in ASYM respect to SYM, *but* not with respect to p53SYM (320 genes); A_down: down-regulated in ASYM and respect to SYM, *but* not with respect to p53ASYM (265 genes); B_up: up-regulated in ASYM with respect to p53SYM, *but* not with respect to SYM (194 genes); B_down: down-regulated in ASYM with respect to p53ASYM, *but* not with respect to SYM (56 genes) (see Fig. 2 also); A_up/B_down: up-regulated in ASYM with respect to SYM *and* down-regulated in ASYM with respect to p53SYM (5 genes); and A_down/B_up: down-regulated in ASYM with respect to SYM *and* up-regulated in ASYM with respect to p53SYM (5 genes). Within the larger SRPA gene set, up-regulated ASRA genes are A_up/B_up; and down-regulated ASRAs are A_down/B_down.

The quantitatively defined ASRA gene subset is not exhaustive for DSC biomarker candidates. Some members of other subsets of SRPA genes may also prove to be useful DSC biomarkers. Because of the high stringency used for ASRA gene selection, some A_up and A_down genes with qualitatively ASRA character were excluded. *Plxdc2* and *ephb6*, whose mRNAs were undetectable in the SYM and p53SYM states, are examples of such non-ASRA SRPA genes. We performed quantitative real-time RTPCR assays for *plxdc2* and *ephb6* and three up-regulated ASRA genes *cxcr6*, *fst*, and *robo1*. *Plxdc2* mRNA was not detected in the SYM state, but was detected in the ASYM state (n = 3, p = 0.001). The respective fold up-regulation for the other 4 genes, in the ASYM state with respect to the SYM state, was 4.6 (*ephb6*; n = 3, p = 0.04); 8.6 (*cxcr6*; n = 6, p = 0.002; reported previously in [30]); 6.5 (*fst*; n = 3, p = 0.007); and 8.3 (*robo1*; n = 3, p = 0.10).

### Evaluation of the ASRA gene subset as a signature for specifying self-renewal pattern

All SRPA genes are potential participants in mechanisms that control DSC self-renewal pattern, IMPDH II-dependent regulation present in the p53SYM state (*e.g.*, B_up and B_down genes in particular), and nonrandom sister chromatid segregation that occurs during the ASYM state [20], [21]. However, the ASRA gene subset is predicted to be more highly related to DSC asymmetric self-renewal *per se*. As such, it might be used to specify the self-renewal pattern character of isolated tissue cell populations. For this purpose, we developed a bioinformatics approach to evaluating the ability of the ASRA gene subset to serve as a signature for distinguishing asymmetric self-renewal from symmetric self-renewal.

**Figure 4:**
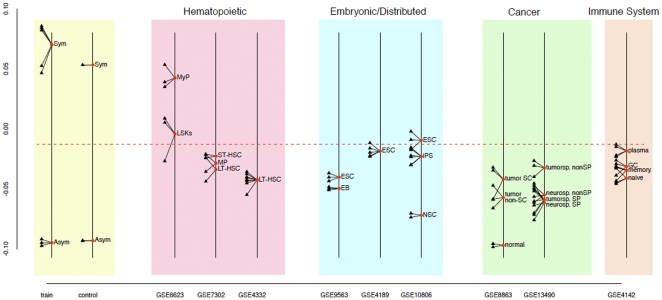
Self-renewal pattern specification by Between Group Analysis (BGA) using the ASRA gene signature. A self-renewal pattern classification field was developed based on BGA training with the original 8 gene microarray datasets (**train**; **SYM** = SYM and p53SYM). The horizontal dashed line delineates symmetric self-renewal character (**upper field**) and asymmetric self-renewal character (**lower field**). The specification ability of the method was tested with independently developed microarray datasets for the ASYM and SYM states (**control**). Hematopoietic DSC-enriched populations include, **LSK**, **ST-HSC**, **LT-HSC**, and **MP** (multipotent progenitors); **MyP**, committed myeloid progenitors. **ESC**, embryonic stem cells; **EB**, embryoid bodies; **iPS**, induced pluripotent stem cells; **NSC**, neural stem cells. Cancer cell populations include mouse mammary cancer derivatives (**left**) and mouse glioma derivatives (**right**): **SC**, stem cells; **normal**, normal mammary epithelial cells; **tumorsp**, tumorsphere; **neurosp**, neurosphere; **SP**, side population. Immune cells included, **plasma** cells; **GC**, germinal center cells; **memory** B-cells; and **naïve** B-cells. **GSE**, Gene Expression Omnibus dataset deposit number.

As shown in Fig. 4, when the 85 gene ASRA gene subset is used as a gene signature in Between Group Analysis (**BGA**) [31], [32], a supervised classification method, with the original gene microarray data as a training set (Fig. 4, train), it provides excellent discrimination of the ASYM datasets from the SYM and p53SYM datasets. Independently developed microarray datasets for cells grown under standard ASYM versus SYM conditions were well discriminated using the signature (Fig. 4, control).

**Figure 5:**
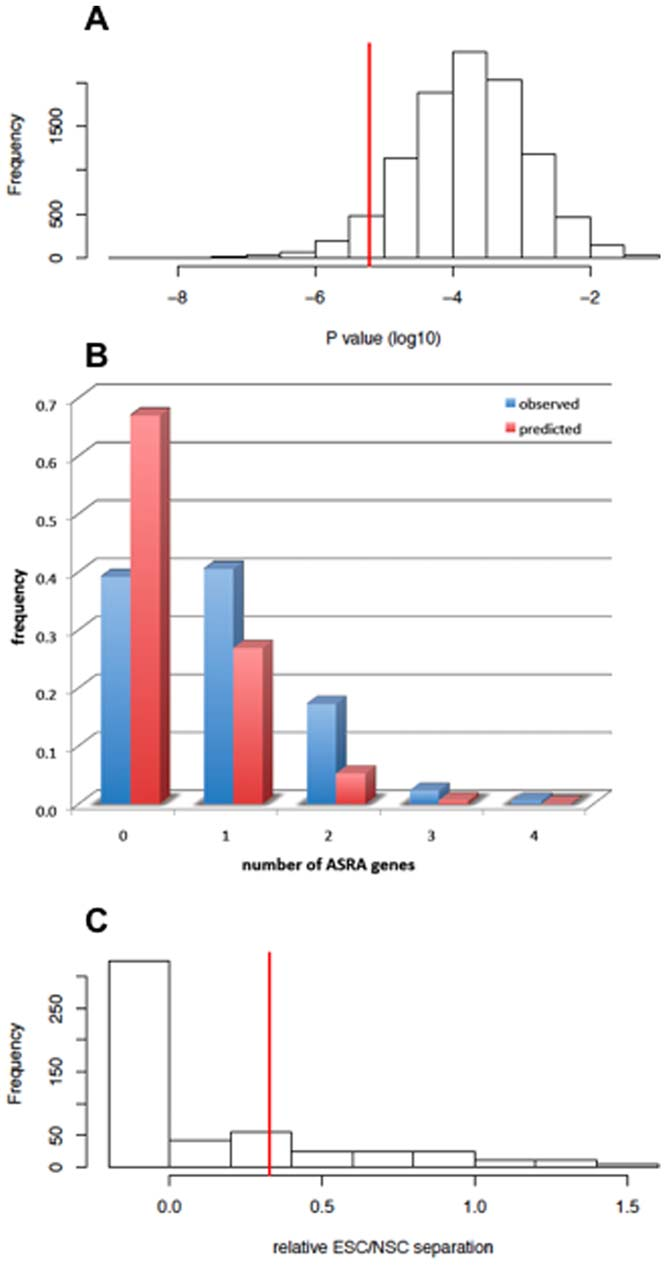
Statistical evaluation of the biological uniqueness of the ASRA gene signature. **A.** Shown is the histogram for p values determined by the Student's t-test for Between Group Analysis (**BGA**) separation of the ASYM *versus* the SYM/p53SYM training datasets described in Fig. 4. Each of the 10,000 determinations was performed with the expression values of a random sample of 85 genes. The **vertical line** indicates the p value of the 85 gene ASRA gene signature. **B.** Comparison of the observed frequencies (**ordinate; blue**) of randomly sampled 85 gene subsets, which had lower p values than the ASRA gene signature in A and contained the indicated number of ASRA genes (**abscissa**), to the frequencies predicted by chance (**red**). **C.** Analyses of the BGA separation distance between microarray data sets for mouse embryonic stem cells (**ESC**) *versus* cultured mouse neural stem cells (**NSC**) relative to the separation distance of respective SYM versus ASYM training datasets (see Fig. 4) using the randomly sampled 85 gene subsets that had lower p values than the ASRA gene signature in A. All gene subsets whose BGA analyses lacked altogether the respective correspondence between ESC∶NSC and SYM∶ASYM separation were grouped as “<0”. The **vertical line** indicates the relative ESC∶NSC separation using the 85 gene ASRA gene signature.

We developed a statistical method to evaluate how unique the ASRA gene signature was for its ability to discriminate known asymmetric self-renewal states from known symmetric self-renewal states based on its ASYM∶SYM∶p53SYM selection design. We used BGA to estimate the probability of finding, by random sampling within the founding microarray data, other 85 gene subsets of greater ability to discriminate self-renewal pattern compared to that of the ASRA gene signature. The Student's t-test was used to determine statistical confidence levels for the degree of ASYM *versus* SYM/p53SYM discrimination. The distribution for 10,000 randomly sampled 85 gene sets with respect to BGA p value is shown in Fig. 5A. Based on this analysis, the ASRA gene signature was in the 5^th^ percentile for the statistical confidence of its ASYM *versus* SYM/p53SYM discrimination, indicating a high degree of biological specificity.

Qualitatively and biologically, the ASRA gene signature is even more unique than its quantitative 5^th^ percentile ranking indicates. Closer inspection of the 515 randomly sampled gene subsets that discriminated with higher statistical confidence shows that they are over-represented for ASRA genes. Fig. 5B indicates that the number of ASRA genes found in the other gene subsets is greater than expected by chance. Another strong indication of the uniqueness of the ASRA gene signature was noted when the 515 higher ranking randomly sampled gene subsets were evaluated for their degree of discrimination of independent microarray datasets for mouse embryonic stem cells (**ESCs**) and cultured mouse neural stem cells (**NSCs**). Based on their stem cell type (*i.e.*, embryonic *versus* distributed, respectively), *a priori*, ESCs and NSCs are predicted to be discriminated by the ASRA gene signature as symmetrically self-renewing and asymmetrically self-renewing, respectively. This distinction is observed in the BGA analysis using the ASRA gene signature (Fig. 4, GSE10806). In contrast, the vast majority of randomly sampled gene subsets did not make this distinction at all; and only 22% gave greater separation of NSCs and ESCs datasets relative to their separation of respective ASYM versus SYM training microarray datasets (Fig. 5C). These analyses indicate that the ability of the ASRA gene signature to discriminate ASYM *versus* SYM self-renewal states is not likely to have occurred by chance, but is more likely to reflect the unique biological design employed for its discovery.

### Use of the ASRA gene signature to specify the self-renewal pattern character of tissue cell preparations

We used the BGA method to specify and quantify the self-renewal pattern character of a variety of cultured and uncultured murine tissue cell populations based on their ASRA gene signatures found in microarray datasets in the Gene Expression Omnibus (**GEO**) repository (see Fig. 4). A striking feature of this analysis was that, overall, for the cell populations considered, most exhibited greater asymmetric self-renewal character (Fig. 4, below dashed line) than symmetric self-renewal character (Fig. 4, above dashed line). The exceptions to this characteristic were one example each of embryonic stem cells, myeloid progenitor-enriched cells (**MyP**), and hematopoietic stem cell-enriched (**LSK**: lineage-negative, Sca-1+, c-Kit+) populations.

Hematopoietic stem cell (**HSC**)-enriched populations were generally specified to have greater asymmetric self-renewal character (GSE7302; GSE4332) [33]. Even in the case for which an HSC-enriched population was specified as symmetric, it showed significantly less symmetric self-renewal character than compared populations enriched for committed myeloid progenitors (GE6623; p = 0.021) [34]. In another dataset entry (GSE7302), populations enriched for multipotent progenitors (**MP**), long-term (**LT**)-HSCs, and short-term (**ST**)-HSCs, based on respective differences in CD34 and FLT3 expression, were not significantly different for asymmetric self-renewal character. Highly-enriched LT-HSCs isolated for young animals at 2–3 months of age or old animals at 22–24 months of age were not significantly different for degree of asymmetric self-renewal character (GSE4332).

Among the cultured cell populations considered, NSCs (cultured alternately on gelatin or as neurospheres), a type of DSCs, had the greatest asymmetric self-renewal character (GSE10806) [35]. Primary NSC cultures had significantly greater asymmetric self-renewal character than mouse induced pluripotent stem (**iPS**; GSE10806) [35] cells and mouse ESCs (GSE10806, GSE4189, GSE9563) [35]–[37], the latter that extended into the symmetric self-renewal zone (p<0.002; GSE10806) [35]. The iPS cells in this comparison (GSE10806) were developed by introduction of Oct4 and Klf4 into the NSCs. Consistent with the idea that differentiation of ESCs may produce asymmetrically self-renewing DSCs, 5-day mouse embryoid bodies (**EB**; GSE9563) had significantly more asymmetric self-renewal character than their precursor ESC cultures (p = 0.01).

Recently, others and we demonstrated that the ASRA protein, Cxcr6 (see Table S1), is expressed by human melanoma cancer stem cells [30]. This finding supports the hypothesis that one important origin of cancer stem cells is mutated DSCs [38]–[40], which in some tumors may retain asymmetric self-renewal [41]. To explore this concept further, we evaluated the self-renewal pattern character of different cell fractions from two different mouse tumor models, mammary cancer (GSE8863) [42] and glioma (GSE13490) [43]. Both models are based in p53-null mice. The mammary cancer study used tumors that develop after transplantation of mammary epithelium from p53-null mice into cleared fat pads of congenic wild-type mice; and the glioma study evaluated cells from tumors that develop in mice transgenic for a *verbB* gene driven by an S100β promoter on a p53-null genetic background. Although all evaluated tumor cell preparations showed significant asymmetric self-renewal character, they all also had significantly more symmetric self-renewal character than normal mammary epithelium (**normal**; GSE8863). Cells identified by their lineage-negative/CD24 high/CD29 high immunophenotype as mammary tumor-initiating cells (**tumor SC**; GSE8863) showed greater symmetric self-renewal character than mammary tumor cells without this property (**tumor non-SC**; GSE8863), but the difference was not statistically significant (p>0.20). In the glioma model, tumor-initiating cells were found in a FACS side population (**SP**; GSE13490) fraction of tumorsphere cultures. This fraction had significantly more asymmetric self-renewal character than the non-SP (*i.e.*, non-cancer stem cell) tumorsphere cell fraction (p<0.005; GSE13490); and it was similar in self-renewal pattern character to SP and non-SP fractions of cultured neurospheres that contain normal NSCs.

Because of the long-lived nature of immune system memory cells, we evaluated their self-renewal pattern character. In immunized mice, germinal center cells (**GC**), memory B-cells, and naïve B cells exhibit significantly greater asymmetric self-renewal character than short-lived plasma cells (p<0.032; GSE4142) [44]. These data suggest significant asymmetric self-renewal by long-lived immune cells. Previous investigators have noted that memory B-cells, memory T-cells, and LT-HSCs shared a common transcriptional program [44]. Based on this ASRA gene signature interrogation, we suggest that a significant fraction of their shared gene expression is related to asymmetric self-renewal.

### Asymmetric expression by ASRA proteins and related SRPA proteins

Individually or in combination, proteins encoded by ASRA genes (*i.e.*, ASRA proteins) are predicted to provide a new type of highly specific biomarkers for asymmetric self-renewal. To the degree that asymmetric self-renewal is an exclusive property of DSCs, some ASRA proteins are predicted to be specific and universal biomarkers for DSCs. By our design, ASRA proteins might mark asymmetrically self-renewing DSCs, their non-stem cells sisters, or both. The ideal ASRA protein might be predicted as one that marked only the asymmetrically self-renewing DSC and not its non-stem sister.

We used two related indirect *in situ* immunofluorescence (**ISIF**) cytometry assays to determine expression patterns of ASRA proteins in cells undergoing either asymmetric self-renewal divisions or symmetric self-renewal divisions. The sister pair (**SPr**) assay is based on plating cells at sufficient sparseness, so that sister cells can be identified based on their close proximity [18], [30]. The second assay is based on the use of cytochalasin D (**CD**) to capture divided sister nuclei in a single undivided parental cell for evaluation of their respective expression patterns. The CD assay has the advantage of use at high cell densities, but it depends on protein expression patterns being unaffected by CD treatment. All candidate biomarkers were evaluated with both complementary assays.

**Figure 6:**
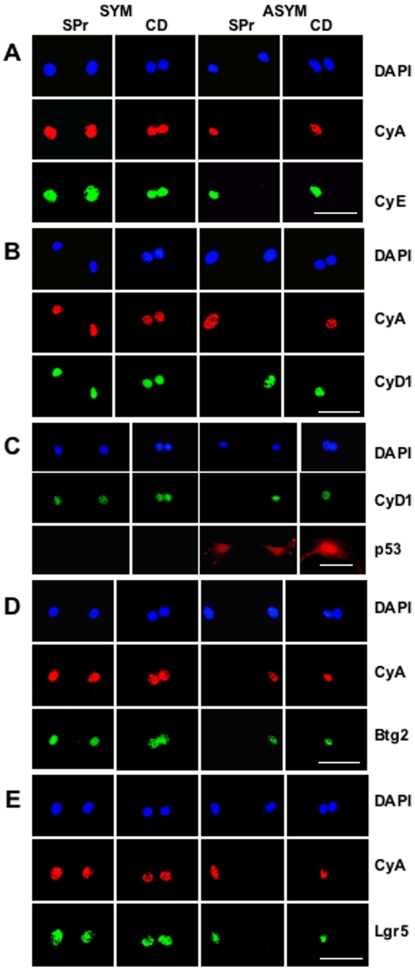
Evaluation of ASRA protein biomarkers by sister pair (SPr) and cytochalasin D (CD) assay. Shown are examples of fluorescent photomicrographs from parallel SPr and CD analyses using dual indirect ISIF performed simultaneously with two different biomarker-specific antibodies of different species origin. Species-specific secondary antibodies conjugated to red and green fluorochromes were used, respectively, for biomarker-specific imaging. **SYM**, symmetric self-renewal by p53-null Con-3 cells with ZnCl_2_. **ASYM**, asymmetric self-renewal by p53-inducible Ind-8 cells with ZnCl_2_ to induce p53 expression. **DAPI**, nuclear DNA fluorescence. **CyA**, indirect ISIF with specific antibodies for cyclin A, an indicator for cycling late G1, S, and G2 cells with greatest expression in G2 phase. **A.** Dual indirect ISIF with antibodies for cyclin A and cyclin E (**CyE**), an indicator for cycling late G1 and S phase cells, with highest expression in early S phase. **B.** Dual indirect ISIF with antibodies for cyclin A and cyclin D1 (**CyD1**), an indicator for cycling G1 or arrested late G1 and early S phase cells, with highest expression in late G1 phase. **C.** Dual indirect ISIF with antibodies for cyclin D1 and **p53**. **D.** Dual indirect ISIF with antibodies for cyclin A and **Btg2**. **E.** Dual indirect ISIF with antibodies for cyclin A and **Lgr5**. See Table 1 for quantitative analyses for the CyA∶CyD1 and CyA∶Btg2 SPr and CD assays. Only the CD analysis was quantified for the CyA∶Lgr5 assays (n = 61, 58; and % co-asymmetric CyA and Lgr5 expression = 20%, 4% for paired nuclei in the ASYM state *versus* the SYM state, respectively; p<0.006 by the two-tailed Fisher's exact test). **Scale bars** = 50 microns.

To validate the SPr and CD assays, we used three well-described cell cycle-specific biomarkers: cyclin A (**CyA**) as a general biomarker for cycling cells, cyclin E (**CyE**) as a biomarker for cycling early S phase cells, and cyclin D1 (**CyD1**) as a biomarker for G1 cells and, more importantly, cells arrested at G1/S of the cell cycle [45]. Fig. 6 provides examples of the major types of SPr and CD expression patterns observed for these biomarkers under conditions of symmetric self-renewal *versus* asymmetric self-renewal with the engineered cell lines. In SPr analyses, all three cell cycle biomarkers showed primarily symmetric expression patterns when the engineered cell lines were cultured under conditions for symmetric self-renewal (Fig. 6A and 6B, SYM, SPr). In contrast, under conditions for asymmetric self-renewal, asymmetric expression patterns increased significantly (Fig. 6A and 6B, ASYM, SPr; see also Table 1, CyA∶CyD1, SPr assay). As predicted, CyD1, the biomarker for non-cycling non-stem sisters, was consistently reciprocally asymmetric with respect to the cycling cell biomarkers (Fig. 6B, ASYM, SPr). Similar relationships were observed in parallel CD assays (Fig. 6A and 6B, CD; see also Table 1, CyA∶CyD1, CD assay), validating this second assay for use with cyclins A, E, and D1. Simultaneous detection of CyA and CyD1 was particularly effective for detecting and quantifying asymmetric self-renewal and symmetric self-renewal (Fig. 6B, ASYM; Table 1, CyA∶CyD1). When p53 was induced, it was always expressed in both sister cells or sister nuclei, despite their clear phenotypic differences (Fig. 6C, ASYM; compare p53 and CyD1). Thus, although the shift to asymmetric self-renewal from symmetric self-renewal by the engineered cells is initiated by p53 expression, it is not due to asymmetric expression of p53 between sister cells.

**Table 1:** Test Quality Metrics for SPr and CD Assays for Asymmetric Self-Renewal.

Assay	% ASYM Pattern		n	p	Sen(%)	Spe(%)	PPV(%)
	Condition						
	ASYM	SYM					
**CyA∶CyD1**							
SPr assay	25	3	61,60	<0.0002	44	94	88
CD assay	25	2	63,60	<0.0001	43	97	94
**CyA∶Btg2**							
SPr assay	47	6	60,50	<10^−6^	58	93	90
CD assay	43	5	53,61	<10^−6^	64	93	88
**CyA∶H2A.Z**							
SPr assay	43	5	58,57	<10^−6^	69	93	89
CD assay	37	3	63,59	<10^−6^	53	95	92

**% ASYM Pattern**, percent of evaluated sister cell pairs or sister nuclei pairs showing an asymmetric protein expression pattern; **ASYM**, under conditions that promote asymmetric self-renewal; **SYM** under conditions that promote symmetric self-renewal; **n**, number of pairs randomly evaluated under each condition for two independent assays; **p**, Fisher's exact test for the statistical confidence of specific asymmetric pattern detection vs. symmetric pattern detection; **Sen**, test sensitivity; **Spe**, test specificity; **PPV**, test positive predictive value.

To date, we have evaluated 6 ASRA proteins and two related SRPA proteins for their expression pattern during asymmetric self-renewal. Plexin domain containing 2 (Plxdc2) protein; glycoprotein (transmembrane) nmb (Gpnmb); KH domain containing, RNA binding, signal transduction associated protein 3 (Khdrbs3); and alpha B crystallin (Cryab) showed symmetrically up-regulated expression in both the asymmetrically cycling stem-like cell and its non-cycling sister (data not shown).

The 4 remaining proteins had distinctive asymmetric expression characteristics during asymmetric self-renewal. Two, cytokine (C-X-C motif) co-receptor Cxcr6 [30] and antiproliferative B-cell translocation gene 2 (Btg2), were originally identified as ASRA genes (see Table S1). The other two, the histone H2A variant H2A.Z and the leucine-rich repeat-containing orphan G protein-coupled receptor 5 (Lgr5), were discovered because of their connection to non-ASRA SRPA genes. H2A.Z itself is an A_down gene (“H2afz” in Table S1) that is consistently reduced in expression during asymmetric self-renewal [46]. Expression of *lgr5* mRNA did not differ significantly among the three self-renewal states. However, its family member *lgr6* was identified as an A_up gene. In transgenic mouse reporter gene knock-in studies, Lgr5 and Lgr6 were previously defined as specific biomarkers for hair follicle and gastrointestinal epithelial stem cells [7], [10], [11].

The asymmetric expression of Cxcr6 during asymmetric self-renewal was reported earlier [30]. Btg2 shows a very similar asymmetric expression pattern limited to the nucleus of the cycling stem-like sister during asymmetric self-renewal (Fig. 6D, ASYM, Btg2). For both proteins, their specific expression in the cycling stem-like cell was confirmed by their co-expression with asymmetrically expressed cyclin A (*e.g.*, Fig. 6D, ASYM, CyA). This feature has been also been confirmed for H2A.Z with the engineered cell lines (data not shown; Table 1) and with *ex vivo* expanded mouse hair follicle stem cells (Huh and Sherley, unpublished data). Fig. 6E data show that asymmetrically self-renewing engineered cells express nuclear Lgr5 in a similar asymmetric pattern. The cycling sister, which retains the stem cell phenotype, expresses Lgr5 (Fig. 6E, ASYM; compare Lgr5 to CyA).

Detection of asymmetrically self-renewing cells using the biomarker pairings of CyA∶CyD1 (reciprocally asymmetric), CyA∶Btg2 (co-asymmetric), and CyA∶H2A.Z (co-asymmetric) was highly significant (see Table 1). We evaluated the quality of each of these combinations as tests for detecting asymmetrically self-renewing cells. For this analysis, we modeled that the asymmetric self-renewal rate of the engineered cell lines under SYM conditions was 0% and under ASYM conditions 100% (previous time-lapse microscopy determined values were 9% and 72%, respectively) [17]. Based on this simplified model, the test quality values in Table 1 were calculated. All three biomarkers were similarly highly specific for detecting asymmetric self-renewal with respect to symmetric self-renewal. However, the CyA∶Btg2 and CyA∶H2A.Z based assays were more sensitive than CyA∶CyD1 based assays, indicating that they scored more than just the cell cycle arrest of the non-stem cell sister. Indicative of their generally high level of specificity, all three assays had a high positive predictive value, meaning that when an asymmetric self-renewal call was made it had an 88–94% chance of being correct.

## Discussion

### Identification of ASRA genes, a subset of SRPA genes

Genetically engineered, p53-inducible murine embryo fibroblasts that model DSC asymmetric self-renewal are the foundation for the development of this unique stem cell gene expression profile resource. Since their original description [25], others and we have reported several specific examples of evidence for their relevance to functions of DSCs *in vivo*. These include the use of molecular pathways discovered in them to expand different types of DSCs *ex vivo* [18], [27]–[29], [47] and the demonstration that they undergo DSC-specific non-random sister chromatid segregation [20], [21]. A recent study also implicated p53 in the regulation of the self-renewal pattern of mouse mammary DSCs and mammary cancer stem cells. In these stem cells, the p53 null genotype was associated with increased symmetric self-renewal at the expense of asymmetric self-renewal [48]. These findings were anticipated by our proposal of p53-dependent regulation of self-renewal pattern as a model for the self-renewal kinetics of DSCs [21], [23].

Our report here that the genetically engineered cell lines induce the *Lgr6* gene and express Lgr5 protein asymmetrically during asymmetric self-renewal further increases confidence that they share important functional properties with DSCs *in vivo*. However, the one previous study of *in situ* detection of Lgr5 by immunohistochemistry in human tissues did not report nuclear expression of the protein [10]. We observed both nuclear and cytoplasm Lgr5 protein in mouse hair follicle stem cells, but only the nuclear form showed asymmetric expression; and detection of both forms is prevented by blocking the Lgr5 antibodies with their peptide antigen (Huh and Sherley, unpublished). Only nuclear Lgr5 was detected in the engineered cell lines. These differences may reflect species and cell type-specific differences in Lgr5 subcellular localization. We note that nuclear localization of other G-coupled protein receptors has been reported for other cell types [49].

Subsets of ASRA genes and the larger set of SRPA genes are also predicted to function in mechanisms responsible for asymmetric self-renewal and closely associated non-random sister chromatid segregation [20], [21]. For example, H2A.Z was described previously for its ability to prevent p53-dependent induction of the cyclin inhibitor CDKN1A [50]. The observed restriction of its expression to the asymmetrically cycling stem cell sister (Table 1) might prevent the initiation of a terminal cell cycle arrest program by CDKN1A that occurs in the non-stem sister. More recently, we have shown, in both the engineered cell model and *ex vivo* expanded mouse hair follicle stem cells, that asymmetric H2A.Z expression is also restricted to the set chromosomes that contain the oldest DNA strands during non-random sister chromatid segregation (*i.e.*, the “immortal DNA strands,”) (Huh and Sherley, unpublished).

There are many published reports of p53-responsive gene signatures [51]–[55]. However, earlier studies were not conducted with cell models that undergo well-defined asymmetric self-renewal; and they were not designed to identify genes specifically associated with this special property of DSCs. Thus, this is the first report of a p53-related gene resource with potential for specifying DSCs exclusively and universally; but, not surprising, a number of ASRA genes were previously described as p53-responsive genes. Several of the most highly expressed ASRA genes are well-known p53-responsive genes (Table S1; *trp53inp1*, *cdkn1a*, cnng1, and *mdm2*). These highly p53-dependent genes score as ASRA genes because their expression level is reduced significantly when asymmetric self-renewal is prevented by transgenic IMPDH II expression, though not always as low as their expression level in the absence of p53 expression. Other ASRA genes are more recently defined p53-responsive genes. Btg2 induction was shown to be required for p53 suppression of murine embryo fibroblast transformation by *ras* [56]. The small heat shock chaperone Cryab was recently discovered as a p53 transcriptional activation target that binds to p53 and regulates p53-dependent apoptosis [55]. Because of their ASRA gene expression pattern, each is now identified for greater potential to identify DSCs and to play important roles in DSC asymmetric self-renewal and non-random sister chromatid segregation.

### Evidence for determined asymmetric self-renewal by individual DSCs

Although asymmetric self-renewal is a defining concept for DSCs, its exact cellular details remain poorly defined. Generally, asymmetric self-renewal by DSCs is inferred retrospectively from their long-term production of lineage-specific differentiated cells in stem cell-deficient hosts. Prospective analyses of asymmetric self-renewal are infrequent because of the technical challenge presented by its dynamic and cell-heterogeneous nature. In fact, even the form of asymmetric self-renewal by DSCs *in vivo* has persisted as a matter of uncertainty since the formulation of the tissue stem cell concept [19], [57]. Stochastic forms, based on multiple stem cells per tissue unit, are at one extreme; whereas tissue units based on single determined stem cells are at the other [58]–[60]. Determined asymmetric self-renewal by individual DSCs is accomplished by divisions that yield a DSC sister and a lineage-committed sister [3], [19].

Two labs [18], [61], including this one, have demonstrated determined asymmetric self-renewal by individual cells in DSC-enriched cultures. In addition, we genetically engineered the described murine cells that individually undergo determined asymmetric self-renewal under experimental control [17], [20], [21], [23], [24]. These lines were the foundation for the development of the presented ASRA gene expression profile resource; and they provided an important orthogonal evaluation of their DSC biomarker potential based on their expression pattern between sister cells from asymmetric self-renewal divisions.

The BGA studies (Fig. 4) provided a first *in vivo* indication of the potential DSC discriminating power of the ASRA gene signature by using publicly available microarray databases for cultured and uncultured murine tissue cells. Because of the well-described marked variability in microarray datasets among different labs and experiments, caution must be exercised in the weight placed on any individual dataset's ASYM *versus* SYM specification. Greater weight can be placed on the relative self-renewal pattern assignments of datasets compared in the same experiment. Although limited by the purity of the examined cell populations and the precision of their deposited microarray analyses, these studies were particularly informative. We found that cell populations enriched for hematopoietic stem cells, one of the best-defined DSC types, had significant asymmetric self-renewal character. This finding provides new evidence that deterministic asymmetric self-renewal is a specific property of DSCs, including uncultured ones.

The BGA studies also illustrate how the ASRA gene signature provides a resource for specifying and quantifying the self-renewal pattern character of cell populations of interest. By using human gene probes that correspond to the mouse ASRA gene signature, similar classifications could be performed for human cell populations of interest [46]. The value of such capability can be appreciated from this report. Compared to the asymmetric self-renewal character of DSC types (*i.e.*, HSCs and NSCs), lineage-committed progenitors, mouse ESCs, and iPSCs each had ASRA gene signatures more characteristic of symmetric self-renewal. This relationship could account for the failure of earlier gene profiling studies to identify “stemness genes,” when ESCs and adult stem cells (*i.e.*, DSCs) were treated as equivalent [62]. Many important stemness genes expressed specifically in DSCs were most likely overlooked, because they were not also expressed significantly in ESCs.

### The uniqueness of the ASRA gene signature

We developed a random sampling-BGA method for evaluating the uniqueness of the ASRA gene signature's ability to discriminate between asymmetric and symmetric self-renewal states (Fig. 5). This method also constitutes a general bioinformatics approach to mining for gene signatures of prescribed gene number that have the greatest statistical power for delineating two biologically distinct cell states of interest. It is noteworthy that, whereas many gene subsets identified by this method distinguished the experimental ASYM and SYM states with greater statistical confidence than the ASRA gene signature (515 out of 10,000 trials; Fig. 5A), only a small fraction of these was also able to discriminate respective NSCs and ESCs as well (22%; Fig. 5C). These relationships support the starting premise that asymmetric self-renewal *per se* is the gnomonic for DSCs [3]. That is to say that, although other gene subsets can be readily found that distinguish the experimental cells in their respective ASYM and SYM states, the ASRA gene signature, defined *a priori* to distinguish self-renewal pattern specifically, is better than most when it comes to discriminating between natural stem cell types with respect to their pattern of self-renewal.

### ASRA proteins and SRPA proteins as potential specific and universal DSC biomarkers

Three ASRA proteins investigated so far (H2A.Z, Cxcr6, Btg2) have ideal asymmetric expression patterns for exclusive and universal identification of asymmetrically self-renewing cells. However, they have another feature that will require more investigation before its effect on DSC biomarker capability can be determined. They are also expressed in both sisters during symmetric self-renewal by the engineered cell lines (*e.g.*, Fig. 6D and 6E) and mouse hair follicle stem cell strains (Huh and Sherley, unpublished). Although this feature models well the concept of DSC symmetric self-renewal, there is no design feature of the engineered cell model *per se* to preclude similar symmetric expression by transient amplifying cells in tissues. Our initial tissue analyses (Huh and Sherley, unpublished) and public databases of immunohistochemical analyses [63] indicate that this shortcoming applies to H2A.Z, but not Btg2. Moreover, Cxcr6, which is expressed in both the nuclei and cytoplasm of the engineered cell lines, shows a high level of nuclear expression only in the cycling stem-like cell of asymmetric divisions. During symmetric divisions, the nuclei of both sister cells show a marked reduction in nuclear Cxcr6 [30]. Cxcr6 has not been evaluated in tissues as yet, but this pattern of expression predicts that it will be highly specific for asymmetrically cycling DSCs. Already it has been shown to identify a discrete subpopulation of human melanoma cancer stem cells that produce more aggressive tumors than melanoma cancer stem cells selected by other biomarkers [30].

So far, an ASRA protein expressed only by the asymmetrically cycling DSC-like sister and neither by its asymmetric non-stem sister nor symmetrically cycling sisters in general, has not been identified. Such biomarkers, if found, might provide even more specificity for detecting tissue DSCs. Of course, existing ASRA proteins like Btg2, which appear to be expressed by only rare positive cells in most tissues [63], may prove to be more sensitive biomarkers by recognizing DSCs both during predicted common homeostatic asymmetric self-renewal and rarer symmetric self-renewal. Thus, full utilization of this resource by the stem cell research community may yield the biomarkers needed to move DSC detection from a qualitative discipline to a quantitative one, while simultaneously revealing more of the rich secrets of these remarkable tissue cells.

## Materials and Methods

### Cells

Engineered p53-null vector control line Con-3 (SYM), Zn-dependent p53-inducible line Ind-8 (ASYM), and c-IMPDH II line tI-3 (c = constitutive; p53SYM) were maintained as previously described [21].

### Oligonucleotide microarray development

Whole genome expression profiles of p53-induced Ind-8 cells (ASYM), p53 null Con-3 cells (SYM), and p53 induced c-IMPDH II tI-3 cells (p53SYM) grown for 48-hours in ZnCl_2_-supplemented culture medium were compared by analyzing Affymetrix mouse whole genome GeneChip® 430 2.0 arrays (Affymetrix, Inc. Santa Clara, CA). Three independent preparations of Ind-8 and Con-3 cells and two of tI-3 cells were developed as outlined below.

Ind-8 and Con-3 cells were grown over a 3-day period to about 50% confluency, trypsinized, and replated in zinc-free medium (DMEM, 10% dialyzed fetal bovine serum [**DFBS**], 5 µg/ml puromycin) at a cell number∶plating area∶medium volume ratio of 10^5^∶75 cm^2^∶20 ml. This initial ratio was held constant for all experiments. C-IMPDH II line tI-3 cells were grown and replated in the same way as Ind-8 and Con-3 cells except for the medium (DMEM, 10% DFBS, 5 µg/ml puromycin plus 1 mg/ml G418 sulfate). Sixteen to 24 hours later, the culture medium was replaced with the same volume of respective medium containing 65 µM ZnCl_2_. This time was designated as time = 0; and a set of replicate cultures for each condition (ASYM, SYM, p53SYM) were harvested by trypsinization and counted with a Model ZM Coulter electronic cell counter for later self-renewal kinetics verification. After 48 hours of culture in the Zn-supplemented medium, all cells were harvested by trypsinization and counted to verify their respective self-renewal kinetics (ASYM or SYM) based on comparing calculated asymmetric and symmetric population division cycles (**PDC**) [20].

Total RNA samples were extracted using the Trizol reagent (Invitrogen, Carlsbad, CA) and followed by a clean-up step with the Qiagen RNeasy kit (Qiagen, Valencia, CA). Total RNA quality was tested with the Agilent 2100 BioAnalyzer (Agilent Technologies, Palo Alto, CA). Five µg of total RNA was used for cDNA synthesis, and then cDNA was used to make biotinylated cRNA. cRNA was fragmented and hybridized onto the Affymetrix mouse whole genome GeneChip® 430 2.0 array. The arrays were washed and quantified with a fluorescence array scanner. After scanning the arrays, quantification and statistics were performed using model-based expression and the perfect match (PM) minus mismatch (MM) method in the G-COS® software (version 1.0) and/or the dChip software version 2005. Data across all 8 arrays were originally normalized by setting target intensity at 500 in the G-COS.

### Microarray data analysis

Background correction, normalization, and summarization of data were carried out using RMA [64] as implemented in R. A custom chip description file (CDF), which organizes the oligonucleotide probes on the chip based on the latest version of RefSeq, was used [65]. The custom RefSeq CDF results in 22,587 probe sets and can be downloaded as version 12 at: http://brainarray.mbni.med.umich.edu/Brainarray/Database/CustomCDF/CDF_download.asp.

Differentially expressed genes were identified using the Bioconductor package RankProd, which uses the rank products method, a non-parametric method that detects genes that are consistently ranked high in lists of up or down-regulated genes in replicate experiments [66]. A percent false positive (pfp) cutoff of 0.05 was used. The raw DNA microarray data have been submitted for public access to Gene Expression Omnibus (http://www.ncbi.nlm.nih.gov/geo/) and can be obtained using the following accession number: GSE25334. All data are minimum-information-about-a-microarray-experiment (MIAME) compliant, as detailed on the MGED Society website http://www.mged.org/Workgroups/MIAME/miame.html.

### Between group analysis

Genes identified as differentially regulated in both the Asym∶Sym and the Asym∶p53Sym comparisons (the ASRA genes) were used in supervised classification of additional samples using Between Group Analysis (BGA), as implemented in the R package MADE4 [32]. Correspondence Analysis was used to ordinate the training set of the three asymmetrically and five symmetrically self-renewing samples. Independently developed control samples from our own laboratory and test samples publicly available from GEO were then classified by projecting them onto the discriminator axis.

### ASRA protein expression pattern analyses

Specific indirect ISIF conditions for sister pair assays and cytochalasin D assays for cyclin A and Cxcr6 have been described [30]. Cyclin D1, cyclin E, p53, Btg2, H2A.Z, and Lgr5 indirect ISIF analyses were performed with the following respective antibodies: rabbit anti-cyclin D1 monoclonal antibody (Abcam, Inc.) at a 1∶100 dilution; rabbit anti-cyclin E polyclonal antibodies (Abcam, Inc.) at a 1∶500 dilution; mouse anti-p53 monoclonal antibodies (Abcam, Inc.) at a 1∶500 dilution; rabbit anti-Btg2 polyclonal antibodies (Santa Cruz Biotechnology, Inc.) at a 1∶200 dilution; rabbit anti-mouse H2A.Z polyclonal antibody (Cell Signaling Technology, Inc.) at a 1∶200 dilution; and goat anti-mouse Lgr5 polyclonal antibody (Santa Cruz Biotechnology, Inc.) at a 1∶50 dilution. As required, AlexaFluor 568 goat anti-rabbit IgG (H+L) (1∶300 dilution), AlexaFluor 568 goat anti-mouse IgG (H+L) (1∶500 dilution), and AlexaFluor 488 goat anti-rabbit IgG (H+L) (1∶300 dilution) were used as secondary detection antibodies (Invitrogen, Inc.).

## Supporting Information

Table S1 Self-renewal pattern associated (SRPA) genes, including the 85-gene asymmetric self-renewal associated (ASRA) gene signature. Expression data and annotation of all genes found to be significantly differentially regulated by the described RankProd intersection analysis is presented (See *Materials and Methods*). A RankProd percent false positive (**pfp**) cutoff of 0.05 was used. The expression values are given as log2 values. The fold change (**FC**; based on log2 values) refers to the average ratio of probe intensities on the ASYM chips to corresponding probe intensities the SYM chips. Genes are grouped based on the comparison in which they are differentially regulated. Genes up-regulated in the “A” (ASYM *vs.* SYM) *and* “B” (ASYM *vs.* p53SYM) comparisons are listed as ASRA_up; those that are down-regulated in these two comparisons are listed as ASRA_down. Genes that are up-regulated in only one comparison are listed in the A_up or B_up sections, and conversely genes that are down-regulated in only one comparison are listed in the A_down or B_down sections. Some genes are up-regulated in one comparison, but down-regulated in the other (*i.e.*, if SYM>ASYM>p53SYM or if p53SYM>ASYM>SYM). These appear in the last two sections. Detection calls – whether expression is “present,” “marginal,” or “absent” (**P**, **M**, or **A**) – are summarized in columns immediately following the log2 expression values. If the call is A for all replicate chips for a self-renewal condition, the value cells are shaded; and if the call is P for all chips, the value cells are unshaded. If the calls are all M, or if they differ among replicate chips, an exclamation mark is given followed by the individual replicate calls. Data on the localization of the gene products was obtained from LOCATE [1]. “Type I” indicates that the protein is predicted to have one transmembrane domain, but does not have a signal peptide; and “type II” indicates that the protein is predicted to have a signal peptide and a transmembrane domain. In some instances, isoforms from the same gene are predicted or known to have different subcellular localizations. In these cases, multiple subcellular localization indicators appear.
(XLS)
